# Perceptions of Dutch nurses carrying methicillin-resistant *Staphylococcus aureus*: a qualitative study

**DOI:** 10.1186/s12912-020-00441-x

**Published:** 2020-06-10

**Authors:** Lotte van Heuvel, Renske Eilers, Sabiena G. Feenstra, Manon R. Haverkate, Aura Timen

**Affiliations:** 1 VU University Amsterdam, Amsterdam, The Netherlands; 2 Centre for Infectious Disease Control, National Institute for Public Health and the Environment (RIVM), Bilthoven, The Netherlands; 3 Athena Institute, VU University, Amsterdam, Netherlands

**Keywords:** Methicillin-resistant *Staphylococcus aureus*, MRSA, Carriage, Colonization, Experiences, Impact, Nurses, Healthcare workers, Interviews, Netherlands

## Abstract

**Background:**

Carriers of methicillin-resistant *Staphylococcus aureus* (MRSA) experience a variety of personal and social consequences, despite the asymptomatic nature of carriage. Some of these consequences are inherent to the application in practice of strict infection prevention guidelines. However, the experiences of nurses carrying MRSA have not been documented. This study aimed to describe the experiences of nurses carrying MRSA to get insight into the impact of MRSA carriage on nurses in a country with a “search-and-destroy” policy for MRSA.

**Methods:**

A qualitative study was conducted among eighteen nurses who experienced MRSA carriage and were working in healthcare organizations in the Netherlands (e.g. hospitals, nursing homes and home care). Semi-structured interviews were conducted using an interview guide. The interviews were audio tape recorded, transcribed and analyzed using thematic analysis.

**Results:**

MRSA carriage has an impact on the life of nurses during four distinct phases: becoming aware of carrying MRSA, processing information and guidance, experiencing consequences of carriage and, when applicable, a life after eradication of MRSA. Each phase was found to be associated with negative consequences. The impact of MRSA carriage on the daily life of nurses is mostly influenced by the experience of consequences of MRSA carriage – including a ban to work with patients, eradication treatment with antibiotics, and social isolation from others – despite the asymptomatic nature of MRSA carriage itself. In addition, lack of information and guidance increased the impact of carriage.

**Conclusions:**

This study shows nurses experience various consequences of MRSA carriage, despite the asymptomatic nature of carriage. The work ban, eradication treatment and social isolation influenced the nurses’ work-related future, personal health and social environment. The impact of carriage may be reduced by clear information and guidance, and support from others. Therefore, sufficient information and guidance needs to be given to MRSA carriers by healthcare organizations.

## Background

Antibiotic resistance is a considerable health problem worldwide, resulting in ineffective antibiotic treatment of serious infections [1, 2]. Antibiotic resistance leads to a rise of healthcare costs because of increased treatment costs, prolonged and more frequent hospitalization, and the need for infection control measures [3, 4]. In the Netherlands, infection prevention guidelines are in place to minimize the spread of antibiotic resistance, including a guideline regarding methicillin-resistant *Staphylococcus aureus* (MRSA) as part of the “search-and-destroy” policy for MRSA [5–7]. This policy includes screening of high-risk groups and hospital admission in strict isolation of patients carrying MRSA.

MRSA can cause, among others, skin and soft tissue infections, bloodstream infections and pneumonia [8, 9]**.** However, MRSA carriers often experience no symptoms [10]**.** Still, MRSA carriers experience personal and social consequences. Personal consequences occur on a psychological level because carriage is perceived as a psychological strain [11]. Carriers of antibiotic-resistant pathogens experience an increase in depressive symptoms, which may lead to a decreased quality of life [4]. Furthermore, research from Rump et al. [12] has shown MRSA carriers experience poor mental health as a consequence of MRSA-associated stigma. Social consequences of MRSA are related to restrictions in social life, as MRSA carriers may isolate themselves because they are afraid to infect others [11]. In addition, MRSA carriers experience social isolation in hospitals and nursing homes resulting from the infection prevention guidelines [5, 13].

Dutch healthcare workers carrying MRSA may experience additional consequences of carriage because of the country’s “search-and-destroy” policy for MRSA. As result of this policy, healthcare workers are banned from working with patients during the duration of MRSA colonization [6]. Furthermore, depending on the extent of MRSA carriage, healthcare workers are advised to take antibiotic eradication treatment. Nurses are amongst healthcare workers the ones who have prolonged, direct contact with patients, thus prone to become carriers of MRSA. They are at risk of acquiring colonization or infection with MRSA, for instance due to breaches in infection control precautions or lack of awareness of MRSA colonization among patients [10]. However, the experiences of nurses carrying MRSA have not been investigated properly. This study aimed to describe the experiences of Dutch nurses carrying MRSA to get insight into the impact of MRSA carriage on their daily life.

## Methods

### Study population

This cross-sectional, qualitative study was conducted among nurses working in healthcare organizations in the Netherlands (*n* = 18). The participants were selected using convenience sampling, by placing an online advertisement at three nursing organizations (e.g. website and Facebook of these organizations) and contacting three Dutch hospitals. Contact persons in each hospital were asked to approach employees who experienced MRSA carriage and inform them about this research. Interested nurses were able to contact the researchers. An information letter was sent to the respondents containing details of the study, including the aim and method. Nurses were included in the study if they were confirmed carrier of MRSA – including livestock-associated MRSA (LA-MRSA) – and experienced the infection prevention guidelines [6, 7] for healthcare employees. Participants were included until data saturation was achieved.

The participants consisted of registered nurses (*n* = 15), a practical nurse (*n* = 1) and nursing students (*n* = 2) (Table 1). The nurses differed in age, ranging between 21 to 63 years (mean age, 38 years). They worked in various healthcare organizations throughout the Netherlands, including hospitals (*n* = 15), nursing homes (*n* = 2) and home care (*n* = 1). The nurses were carrier for a period of time between the years of 2005 and 2017. The length of their MRSA carriage ranged between 1 and 2 days to 1.5 years. Two participants were still carrier during the time of the interview.

**Table 1:** An overview of the age, profession and period of carriage of the participants

Participant	Age (years)	Profession	Healthcare organization	Period of carriage	Time of carriage
1	30–39	Registered nurse	Hospital	2016	± 2 weeks
2	40–49	Registered nurse	Hospital	2016	± 2 weeks
3	30–39	Registered nurse	Hospital	2009 – present	> 8 years
4	50–59	Registered nurse	Home Care	2015	± 8 weeks
5	20–29	Nursing student	Nursing Home	2013	± 5 days
6	20–29	Registered nurse	Hospital	2012	± 6 months
7	60–69	Registered nurse	Hospital	2007 - present	> 10 years
8	20–29	Registered nurse	Hospital	2015	± 6–8 weeks
9	30–39	Registered nurse	Hospital	1. 2016	1. ± 5–6 weeks
				2. 2017	2. ± 5–6 weeks
10 ^a^	20–29	Nursing Student	Hospital	1. 2005	1. ± 6 weeks
				2. n.a.	2. n.a.
				3. n.a.	3. n.a
11	20–29	Registered nurse	Hospital	2016	± 5 months
12 ^b^	40–49	Registered nurse	Hospital	2012	± 1 year
13	30–39	Registered nurse	Hospital	2015	± 5 months
14	20–39	Registered nurse	Hospital	2013	± 1–2 days
15 ^a^	30–39	Registered nurse	Hospital	1. 2005	1. ± 4 weeks
				2. 2006	2. n.a.
16	40–49	Registered nurse	Hospital	2016	± 2.5 months
17	50–59	Practical nurse	Nursing home	2006	± 6 weeks
18	50–59	Registered nurse	Hospital	2012–2014	± 1.5 years

^*a*^*multiple times carrier of MRSA*

^*b*^*LA-MRSA*

*n.a.* not available

### Data collection

Semi-structured interviews with open-ended questions were conducted with the respondents in April and May 2017 at a location they preferred (e.g. home or work). The interviews were audio tape recorded and lasted approximately 30 to 80 min. One researcher (LvH) conducted the interviews, using an interview guide for structure. The interview guide was created using the Health Belief Model and Self-Regulation Model of Illness [14–16]. Six themes were discussed during the interviews: (1) MRSA carriage, (2) measures, (3) information, (4) coping with carriage, (5) social environment and (6) looking back at the period of carriage. During the interviews, the participants could express their feelings and experiences regarding MRSA carriage. They received a gift voucher of €10,- after participation. The participants were sent a summary of the interview afterwards for respondent validation.

### Data analysis

The recorded interviews were transcribed verbatim and analyzed applying thematic analysis [17]. The transcripts were analyzed using software program ATLAS.ti (Version 7). The researchers used open coding to derive codes directly from the data, axial coding to identify coding categories and selective coding to select themes among these categories. The coding process of four interviews was discussed with a second independent researcher (RE) until consensus was reached. The researchers analyzed the interviews quickly after data collection, to assess when data saturation was reached.

## Results

**Fig. 1:**
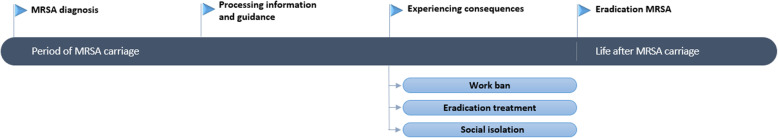
Timeline period of MRSA carriage

The results are described in chronological order in the form of a timeline, starting from the moment of MRSA diagnosis until the interview (Fig. 1). In this time frame, four important moments could be defined: (1) becoming aware of carrying MRSA, (2) processing information and guidance, (3) experiencing consequences of carriage, and when applicable (4) a life after eradication of MRSA. Consequences of carriage include a ban to work as a nurse, eradication treatment with antibiotics, and social isolation from others. The timeline provides insights into what nurses sequentially experienced as MRSA carriers.

### Becoming aware of carrying MRSA

At the moment of diagnosis, the participants reacted differently to the announcement they were tested MRSA positive. For most participants this news was overwhelming, leading to reactions such as sadness, crying, confusion and anxiety about the future, feelings of powerlessness and disbelief about the situation. Most nurses showed signs of panic, for instance: *“I remember that the assistant of the general practitioner called me and told me I had MRSA and I started to cry badly. [ …*] *I was in love with my job and suddenly I could not do that anymore. I had the feeling I could never do it anymore, so I was in a state of panic.” (P6: 6 months carrier).*

The participants were all aware of the work ban for MRSA positive healthcare workers as part of the Dutch “search-and-destroy” policy for MRSA. Consequently, they were afraid never to be able to work as a nurse again. The diagnosis made them think about the cause of their carriage: *“In the beginning I felt guilty that I was a carrier. ‘What did I do with that patient? Did I do something wrong? Did I wear gloves and washed my hands properly?’ You start to rethink how you treated the wound of that patient.” (P9: 2x carrier 5–6 weeks)* However, most nurses did identify MRSA carriage as an occupational hazard with a higher susceptibility for MRSA colonization.

The participants had many questions when diagnosed with MRSA. Most questions referred to social interactions with others, asking simple questions such as “Can I give that person a hand or not?” The diagnosis had an impact on the daily life of the nurses because they felt insecure about their future. One participant mentioned: *“My life was turned upside down completely because I did not know what the future would look like.” (P12: 1 year carrier).*

### Processing information and guidance

#### Lack of clear information

The participants received information from the healthcare organization they worked at concerning MRSA, including rules about working during the period of carriage and treatment to eradicate MRSA. However, most of the participants (*n* = 12) found at least a part of this information unclear. This created feelings of uncertainty. As result of a lack of information, the nurses searched online for additional information about MRSA carriage. This online information resulted in some participants to try foods and supplements they thought might help to eradicate MRSA.

One-third of the participants mentioned they felt they received enough and clear information about, among others, the consequences of MRSA carriage. They expressed this helped them to cope with their carriage. Information also helped to feel at ease: *“How you deal with MRSA carriage is your own problem, but the provision of information prevents a lot of questions, frustration, irritation and confusion.” (P17: 6 weeks carrier)* In particular, information about the future helped the participants to cope with the anxiety and insecurity they experienced during the MRSA diagnosis. The nurses wanted to receive information as soon as possible, preferably at the time of diagnosis, to reduce uncertainty and follow the correct protocol.

#### Guidance helps to cope with carriage

Guidance from other people helped the nurses to cope with their carriage, as they felt they were supported and cared for. The nurses appreciated it if others were willing to listen to their stories. This way, they felt others were able to help them manage their MRSA carriage. Therefore, guidance is an important factor that influences how the nurses experienced their carriage: *“Despite the anxiety and worries you have I think MRSA carriage does not have to be a really bad experience when you are not frightened and receive good guidance.” (P1: 2 weeks carrier).*

However, only a few participants received guidance from their healthcare organization during the period of MRSA carriage. Therefore, guidance and support from family, friends and colleagues was important for the nurses. Nurses who were open about their carriage to others received support. These nurses found it important to share their experiences because they felt people did not understand what they were going through. However, some nurses were reluctant to be open to others, as they were afraid of negative reactions.

### Experiencing consequences of MRSA carriage

#### Banned from working as a nurse

After the MRSA diagnosis, the nurses were immediately banned from working with patients during their period of carriage. Most participants found it difficult to accept the work ban, as they did not experience any physical symptoms of MRSA carriage. The work ban had a big impact on the nurses, because work included a large part of their daily life**.** Part of the participants had to stay at home during their time of carriage (*n* = 8), while others could perform alternative work tasks (*n* = 9) or did not experience any measures (*n* = 1 – carrier for only 1–2 days). The work ban was associated with feelings of sadness: *“I have been trained to be a nurse and that is totally different from working as a secretary. That is fun for a couple of weeks, but then you start to think ‘I do not like this anymore. I want my old job back.’ That is not possible, so that is pretty upsetting.” (P3: carrier since 2009).*

Despite these feelings of sadness, almost all participants understood the need for the work ban (*n* = 14). They agreed it is an important measure to prevent the spread of MRSA to patients. However, four participants were skeptical and felt less accepting about the strictness of the work ban and the Dutch rules in comparison with neighboring countries Belgium and Germany: *“I found it difficult that for instance in Belgium they handle the MRSA policy differently. The protocols are not as strict, so if I had applied for a job in Belgium I would have been hired directly and could have worked as a nurse.” (P3: carrier since 2009).*

The work ban led to insecurity about the future among the nurses, not knowing if and when the MRSA could be eradicated. Majority was afraid to lose their job if the MRSA could not be eradicated. For three participants, insecurity about the future was influenced by the financial consequences of MRSA carriage: *“Your financial future becomes more uncertain and that is wearing on you. That creates a bad feeling, because you think ‘what now.’ It creates worries.” (P13: 5 months carrier).*

#### Health consequences of the eradication treatment

After the nurses were banned from working with patients, they were confronted with a treatment to possibly eradicate the MRSA. The eradication treatment consisted of daily body scrub, oral antibiotics and special attention was given to laundry procedures. The treatment was hardly feasible for the nurses because it was highly time consuming. In addition, the negative consequences of the antibiotic treatment influenced the nurses’ ability to perform all procedures.

Still, all participants were highly motivated to follow the measures correctly, because they were desperate to eradicate the MRSA. In these moments of desperation, most participants tried everything for possible eradication: *“I did everything to get my job back. I would have stood on my head the whole day if that was necessary. I would have gone to the hospital in the middle of the night for something if that was necessary. I would have done whatever it takes.” (P7: carrier since 2007).*

The participants who were treated with antibiotics (*n* = 14) experienced this treatment as “horrible” because of the different health consequences, including gastrointestinal symptoms and fatigue. These consequences influenced the daily life of the nurses, in particular their social life, as some were too sick to attend social activities. As result, the participants were hesitant to undergo antibiotic treatment in the future: *“I would not undergo treatment another time. I think it had a lot of consequences for my health. I think everything is okay now, but up till one year after the treatment I felt less fit.” (P4: 8 weeks carrier).*

#### Social isolation from other people

##### Self-inflicted isolation

As result of their MRSA carriage, most nurses distanced themselves from others. This social isolation created feelings of loneliness. Still, the nurses’ sense of responsibility to protect others outweighed the social consequences of their isolation: *“You feel … excluded is a strong word, but it definitely influences your daily life and social life. But you do not want to hurt someone, so your choice is easy.” (P3: carrier since 2009).*

Some participants made a distinction between contact with healthy and more vulnerable people, such as sick people and elderly. The participants often avoided contact with vulnerable groups to avoid infecting them: *“During my period of carriage I was, and still am, informal caregiver of my grandma. I did not go there in case I would possibly transmit something, as I knew that she got care herself.” (P5: 5 days carrier)* Most participants did remain in contact with healthy people, as they felt these groups experience no severe consequences of MRSA. However, some participants did avoid contact with colleagues, afraid of infecting them and unknowingly transfer MRSA to patients inside the hospital.

##### Isolation as consequence of a stigma on MRSA

The participants experienced stigmatizing reactions from other people. For instance, others isolated themselves from the nurses because they were worried of becoming colonized or infected with MRSA themselves. The nurses noticed people *“literally took a few steps back, like ‘I need to get some distance.’” (P5: 5 days carrier)* This form of non self-inflicted isolation created feelings of loneliness and sadness among the nurses. Some participants did understand people’s adverse reactions towards MRSA carriers, as they understood people could be afraid of becoming a carrier, but most participants felt disappointed and alone when people avoided contact.

Because of the stigmatization, the nurses compared themselves with lepers or aids patients. Several participants (*n* = 7) felt they were “branded” with a mark that they were infectious and contagious. The stigmatization had an impact on the social life of the nurses: *“You are a little housebound. I felt like how lepers lived. I thought ‘oh God it seems I am banned from society.’” (P16: 2.5 months carrier).*

##### Isolation in the hospital as a patient

Five nurses were admitted to the hospital during their period of carriage because of underlying disease and experienced strict isolation procedures.^1^ They mentioned this was a horrible experience. The nurses were able to cope with the isolation through their knowledge of the process, gained by working as a nurse.

In the end, the nurses’ own experience of isolation helped them to empathize with patients. For instance, one participant understood it is annoying for patients that *“you cannot recognize someone because you only see someone’s eyes.” (P11: 5 months carrier)* Consequently, this participant was later more attentive to patients in isolation: *“I think I learned from it. If someone is in isolation you need to go there, despite that you need to put on your suit or a surgical mask. Just quickly ask how that person is doing.” (P11: 5 months carrier).*

### Life after eradication of MRSA

Most nurses still experienced consequences of their MRSA carriage after eradication. The participants remained afraid to become carrier again, resulting in insecurity about the future: *“It has a very long aftermath of insecurity. I had to do MRSA tests till one year after and every time you are still insecure. [ …*] *‘What if I have it again? What if I have to stay at home again?’” (P13: 5 months carrier).*

The duration of the period of carriage influenced the impact of MRSA carriage. A relatively short period of carriage helped the nurses to cope with their carriage, as they could quickly leave this period behind them. However, most nurses needed time to come to terms with their MRSA carriage: *“It took me a very long time to make peace with it. I think people do not completely understand what the impact is on your feelings.” (P13: 5 months carrier).*

As result of the MRSA carriage, the nurses made changes in their daily life. For instance, some participants are now more attentive to taking correct hygiene measures while others are more focused on their health. In addition, some nurses also experienced changes in their daily life on a personal level: “*There is a life before and after this. It really changed me.” (P18: 1.5 years carrier).*

## Discussion

This study provides insight into the impact of MRSA carriage on the daily life of Dutch nurses. The reported impact was related to a lack of information and guidance and experiencing consequences of carriage, including the work ban, eradication treatment and social isolation from others. However, guidance and support from the healthcare organization and social contacts may help the nurses to cope with carriage. In the end, a longer period of MRSA carriage was suggested to have a greater impact on daily life because this prolongs the negative consequences.

From this research it appears that experiencing consequences of carrying MRSA, while experiencing no symptoms, has the greatest impact on the life of nurses. The nurses struggled to accept they could not work with patients and needed antibiotic treatment while they were not sick. They experienced a conflict between two values of medical ethics: the principles of beneficence and non-maleficence (“do no harm”) of the principle-based ethical theory [18, 19]. The nurses found it difficult to outweigh the potential benefits of the work ban for patients and harms of the antibiotic treatment for themselves.

Two consequences of MRSA carriage are persistent throughout the whole period of carriage. First, the nurses experienced recurring insecurity about their future. Insecurity starts at the moment of diagnosis and ends with anxiety for a future period of carriage. Second, nurses are concerned about interacting with other people. They show concern at the start of their carriage and follow this by isolating themselves from others. They continue social isolation during the whole period of carriage. This isolation is related to stigma.

The nurses experienced two forms of stigma: enacted stigma and self-stigma. Enacted stigma is the “perception of actually being treated differently because of MRSA” [12]. The nurses experienced enacted stigma in the form of stigmatizing and discriminating reactions of others. They also experienced self-stigma, which is defined by Scambler & Hopkins [20] as “felt stigma”. Felt stigma refers to a feeling of shame and the “fear of enacted stigma”, which leads people to form negative perceptions towards themselves. Felt stigma relates to the nurses’ self-inflicted isolation. These isolation measures are in accordance with self-stigma of mental illness, where people impose self-isolation as result of self-discrimination [21].

To the best of our knowledge, this is the first study that has focused specifically on the experiences of MRSA carriage among nurses. Some of these experiences are similar to the experiences of MRSA positive patients. One similarity is the feeling of anxiety about infecting others, paired with a feeling of contagiousness and dirtiness [11, 22, 23]. Furthermore, literature shows patients also experience social implications of acquiring MRSA, as patients restrict their interactions with others [24]. Moreover, MRSA patients experience feelings of being an outsider, comparing MRSA with having the plague or leprosy [23–26]. They feel marked and experience stigmatization and discrimination [12, 27] as well as the nurses in our study.

The impact of hospital isolation differs between nurses and patients carrying MRSA. Literature shows the impact of MRSA carriage for patients is mainly influenced by isolation during hospital stay, because they are isolated from others [25, 28]. For nurses, hospital isolation had less impact because of their knowledge as a nurse about the (need for) isolation measures. Social isolation in daily life was more impactful for the nurses.

### Strengths and limitations

One strength of the qualitative approach of this study is that it allows for in-depth exploration of the experiences of the participants [29]. The broad explorative design allowed us to extensively explore the experiences of nurses on MRSA carriage, presumably resulting in data saturation. Respondents in this study were included from different regions of the Netherlands. This resulted in an understanding of the perspectives of nurses working in diverse healthcare organizations, providing insight into different MRSA protocols of organizations and the impact of these protocols. The inclusion of various age groups and durations of carriage further completed the understanding of MRSA carriage among nurses.

However, the participant sample also requires consideration, as convenience sampling resulted in an uneven distribution of sex. In this study, only 1 male and 17 females were included. Nevertheless, males are also underrepresented in healthcare, as approximately 1 out of 10 Dutch nurses are male [30]. Convenience sampling could have also resulted in selection bias and overrepresentation of nurses who experienced a high impact of their MRSA carriage. These nurses were more inclined to react to the online advertisement. However, this study also recruited nurses indirectly through contact persons in hospitals, including nurses who did not experience a high impact of MRSA carriage. Another limitation of the qualitative approach is the possibility of recall bias, since questions were asked concerning past events. Some participants had difficulty remembering specific details regarding their carriage, while other participants could strongly remember everything. This qualitative study was conducted and analyzed mainly by one researcher, which could have impacted the validity of the data. However, the coding system was refined and reviewed by an experienced researcher. A last point for consideration is the inclusion of participants from different type of healthcare organizations, as only two participants worked in nursing homes and one in home care. This led to underrepresentation of nurses working in nursing homes and home care.

### Recommendations

The main findings of this study reveal that clear information and guidance is needed to reduce the impact of MRSA carriage. In addition, the stigma on MRSA carriers needs to decrease**.** Information provision for healthcare workers can, for example, be improved by means of an information folder, focusing on the implications of MRSA carriage in daily life. Nurses in this study suggested such a folder, as is available to patients, would give reassurance in the first moments after diagnosis. Furthermore, to improve guidance, information on the steps to be undertaken following notification of MRSA carriage should be standardized, clearly worded and shared with all professionals involved in monitoring the carriers (e.g. microbiologists, infection control practitioners and occupational health physicians). Stigmatization might be reduced by creating public awareness on MRSA and MRSA carriage, using visually attractive infographics.

## Conclusions

In conclusion, this qualitative study shows the impact of MRSA carriage among Dutch nurses is related to experiencing consequences of carriage, despite the asymptomatic nature of MRSA carriage itself. The ban to work with patients, eradication treatment and social isolation significantly impacted the nurses’ work-related future, personal health and social environment respectively. Healthcare organizations should improve their information and guidance to reduce the impact of MRSA carriage among their employees. The experiences of carriage should be further explored among healthcare workers, as this is the first (qualitative) study focusing on MRSA carriage among nurses.

## Data Availability

The datasets generated and analyzed during the current study are not publicly available due to them containing information that could compromise research participant privacy, but are available from the corresponding author on reasonable request.

## Abbreviations

LA-MRSA: Livestock-associated methicillin-resistant *Staphylococcus aureus*
MRSA: Methicillin-resistant *Staphylococcus aureus*

## Footnotes

1 Strict isolation in the hospital consists of treatment in a separate isolation room. Nurses cannot enter this room without wearing personal protective equipment, including gloves, a long-sleeve gown, surgical mask and a hair cover. (WIP: [Dutch] Hospitals; Strict isolation. https://www.rivm.nl/dsresource?objectid=e4c2c31d-73a4-46bc-870b-6a0786860872&type = org&disposition = inline (2011). Accessed 16 May 2018.)
